# Editorial: Regenerative liver surgery

**DOI:** 10.3389/fsurg.2026.1993956

**Published:** 2026-09-30

**Authors:** Ivan Romic, Gregor A. Stavrou, Branko Bakula, Ante Bogut

**Affiliations:** 1 Department of Surgery, University Hospital Centre Zagreb, Zagreb, Croatia; 2 Department of Surgery, Klinikum Saarbrucken gGmbH, Saarbrücken, Germany; 3 Department of Surgery, Klinicka Bolnica Sveti Duh, Zagreb, Croatia; 4 Department of Gastroenterology, Sveucilisna Klinicka Bolnica Mostar, Mostar, Bosnia and Herzegovina

**Keywords:** FLR (future liver remnant), hypertrophy, liver, regeneration, surgery

The surgical treatment of patients with extensive bilobar liver malignancies has entered a new era. Regenerative liver surgery is an emerging field that has evolved from recent advances in surgical and interventional techniques which allowed inducing or facilitating liver regeneration before major hepatectomy (1). The well-known capacity of the liver to regenerate has been harnessed in the concept of two-stage hepatectomy. However, the idea of preoperative liver hypertrophy induction has driven the development of novel strategies aimed at achieving safer and more extensive hepatic resections.

The development of regenerative liver surgery can be traced back to the 1990s, when portal vein embolization (PVE) replaced portal vein ligation and emerged as an established strategy for inducing hypertrophy of future liver remnant (FLR). Over the following 3 decades, PVE outcomes were extensively evaluated, and despite significant FLR hypertrophy there were some concerns related to a prolonged interval between the first and second stage which resulted in a substantial proportion of patients becoming unsuitable for subsequent hepatectomy due to progression of the disease (2). In the early 2010s, the introduction of ALPPS represented a major advancement in regenerative liver surgery, enabling rapid hypertrophy of the future liver remnant and substantially shortening the interval between the two stages of resection. The initial results of ALPPS were associated with substantial morbidity; however, with increasing experience and technical refinement, morbidity rates became acceptable, particularly when considered in the context of the limited efficacy of systemic chemotherapy and locoregional therapies in patients with extensive or bilobar colorectal liver metastases (CRLMs) (3). The most recent addition to the field of regenerative liver surgery is interventional double embolization also called liver venous deprivation (LVD), which has demonstrated FLR hypertrophy comparable to ALPPS while potentially offering a lower morbidity/mortality. This may be attributed to the absence of surgical trauma associated with partial or complete liver parenchymal transection, as seen in in ALPPS (4).

At the same time, studies demonstrated that liver volume does not necessarily equal liver function. This issue is particularly relevant in patients with chemotherapy-associated liver injury, steatosis, fibrosis, or cirrhosis. Modern functional assessment using magnetic resonance imaging, hepatobiliary contrast-enhanced imaging, hepatobiliary scintigraphy, and indocyanine green (ICG) clearance provides complementary information about the functional reserve of the liver (5). One study from this special issue (Wang et al.) concluded that quantitative imaging biomarkers of liver function based on gadoxetic acid-enhanced MRI might represents an important preoperative evaluation method. The advantage of this particular method would be the easy access and availability. We definitely agree that some functional assessment method should be employed in all major liver resections including the interstage period when FLR hypertrophy is induced. Beyond embolization/ligation of the portal and hepatic veins, other types of vascular manipulation that may influence liver regeneration warrant further investigation. This is illustrated by the study of Guren D et al., in which preoperative portal hypertension was alleviated through splenic artery embolization. The potential impact of hepatic artery chemoembolization on liver regeneration remains controversial and warrants further investigation. Regarding ALPPS and LVD, it remains to see whether one approach will ultimately prevail or whether each technique will find its role in specific clinical indications, which seems more likely. Studies still evaluate technical refinements of these methods focusing mainly on the type of parenchymal transection.

From an oncological perspective, the optimal indications for standard resection, FLR hypertrophy induction and non-surgical methods remain a subject of ongoing debate (6). As demonstrated by He et al. in their meta-analysis, there is evidence suggesting that surgical resection is associated with improved overall survival compared with radiofrequency ablation in appropriately selected patients. Consequently, there is a prevailing tendency to pursue resection whenever technically and oncologically feasible. In this context, strategies for preoperative FLR augmentation may expand the pool of patients eligible for curative-intent surgery and may therefore play an increasingly important role in the multidisciplinary decision-making process.

Beyond these technical advances, there is a growing need for robust prognostic models capable of predicting the likelihood of successful treatment. One such nomogram was proposed by Xiao et al. for patients with hepatocellular carcinoma undergoing liver resection. As larger datasets incorporating patient-, tumor-, and treatment-related variables become available, these predictive models are expected to become increasingly accurate. The integration of advanced statistical approaches and artificial intelligence (AI) may further enhance their predictive performance, potentially enabling more individualized patient selection and treatment planning in regenerative liver surgery. AI will definitely find its place in other aspects of liver surgery and regeneration. AI-based volumetry, automated segmentation, radiomics, longitudinal assessment of hypertrophy, and prediction of postoperative liver function may allow more precise selection of patients for such procedures and better prediction of their response (7).

At the end, we are aware that there is a shift from standard resections to minimally invasive and ultrasound or fluorescence guided parenchymal-sparing techniques. However, if FLR is insufficient such methods rarely provide an adequate solution. Therefore, regenerative liver surgery remains the only curative strategy e.g., in the setting of multiple bilobar liver lesions. The combination of minimally invasive strategies, FLR induction methods**,** functional imaging, fluorescence guidance, and AI-driven decision support may ultimately create a more individualized form of treatment, paving the way for an exciting future in the field of hepatic surgery.
